# Loss-Related Characteristics and Symptoms of Depression, Prolonged Grief, and Posttraumatic Stress Following Suicide Bereavement

**DOI:** 10.3390/ijerph191610277

**Published:** 2022-08-18

**Authors:** Raphaela Grafiadeli, Heide Glaesmer, Birgit Wagner

**Affiliations:** 1 Department of Psychology, Medical School Berlin, Rüdesheimerstraße 50, 14197 Berlin, Germany; 2 Department of Medical Psychology and Medical Sociology, Medical Faculty, University of Leipzig, Philipp-Rosenthal-Str. 55, 04103 Leipzig, Germany

**Keywords:** suicide bereavement, latent class, depression, prolonged grief, posttraumatic stress, loss-related characteristics

## Abstract

(1) Background: The aim of the present study was to examine symptom classes of major depressive disorder (MDD), prolonged grief disorder (PGD), and posttraumatic stress disorder (PTSD) in a sample of suicide-bereaved individuals, while accounting for loss-related characteristics. (2) Methods: A latent class analysis was conducted to identify classes of the suicide bereaved, sharing symptom profiles, in a German suicide-bereaved sample (N = 159). (3) Results: Our analyses revealed three main classes: a resilient class (16%), a class with high endorsement probability for PGD symptoms (50%), and a class with high endorsement probability for combined PGD/PTSD symptoms (34%). Prolonged grief and intrusive symptoms emerged across all classes, while MDD showed low endorsement probability. Our results indicate an association between class membership and time passed since the loss; however, this applies only to the comparison between the PGD and the resilient class, and not for the PGD/PTSD class. (4) Conclusions: Our results may provide information about the predictability of symptom clusters following suicide bereavement. The findings also represent a significant step towards tailoring treatments based on the needs of relevant suicide-bereaved subgroups through a symptom-level approach. Time passed since loss might explain differences between symptom clusters.

## 1. Introduction

Suicide bereavement represents a widely recognized stressor conferring risk for mental disorders and negative social outcomes. The increasing attention on the vulnerability of this population to forming maladaptive emotional reactions has led to the development of several psychological interventions over the past decade [1]. Despite the variety of existing interventions for suicide-bereaved individuals, results from existing efficacy studies indicate only small to medium effect sizes, while the superiority of psychological interventions over unspecific interventions has not been consistently shown [1,2]. The lack of robustness of these effects might be an indication of a not-yet-fully developed understanding of the specific needs of this high-risk population.

According to a population-based study in Canada, nearly half of suicide-bereaved parents develop at least one mental health disorder within two years after the bereavement (Bolton et al., 2013 [3]). A register-based cohort study from Denmark revealed an increased risk for the development of mental disorders in suicide-bereaved spouses when compared to the general population, or spouses bereaved through other means [4]. Research has shown that suicide bereavement is associated with a high risk for the development of emotional disorders such as major depressive disorder (MDD), anxiety disorders, and specifically posttraumatic stress disorder (PTSD) [3,5,6]. Additionally, a high proportion of those bereaved show persisting grief reactions, with increased probability for the development of a prolonged grief disorder (PGD), with prevalence rates ranging between 7% and 10% [7,8,9,10,11]. Additionally, a co-occurrence of these disorders following bereavement is commonly observed [3,12]. Recent estimates on post-loss symptomatology indicate that 63% of bereaved individuals with PGD show co-occurring depression, while 49% show a PTSD comorbidity [13].

The examination of this co-occurrence following bereavement has received increased attention over the past decade. PGD has been included in the ICD-11 [14] and was only recently introduced as a distinct disorder in the text revision of the DSM-5 (DSM-5-TR; APA 2020 [15]). PGD is characterized by elevated and persistent grief following the loss of a significant person while causing functional impairment to the individual. PGD is mainly characterized by a persistent yearning or longing for the deceased individual. Research notes the high relatedness of emotional disorders, especially between PGD, MDD, and PTSD. Despite the close similarities of these disorders, several main differences exist. For example, while PGD and PTSD share symptoms of intrusion and avoidance, the expressions of these symptoms differentiate. While trauma-related avoidance is associated with fear, bereavement-related avoidance is often associated with loss-related aspects such as (positive) memories of the deceased and separation distress. Sufficient insight into the overlap of emotional disorders exists, as well as with regard to their distinctiveness [16,17].

**Figure 1:**
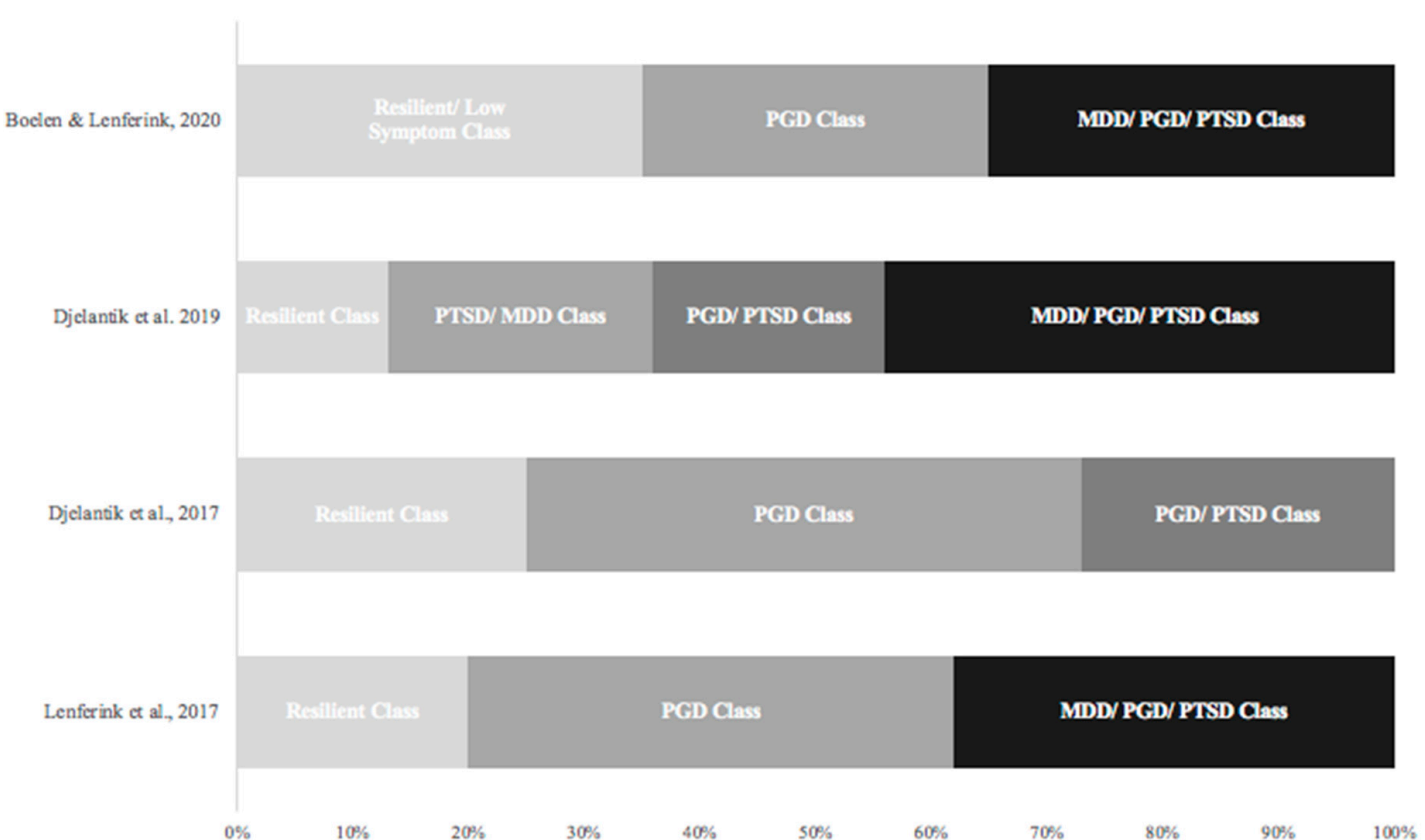
Summary of research findings on symptom profile classes following bereavement. Boelen & Lenferink, 2020 [21], Djelantik et al., 2017, 2019 [18,19], Lenferink et al., 2017 [20].

Following a person-centered approach using latent class analysis (LCA) research on psychological sequelae following bereavement, researchers examined classes of patients presenting similar symptom profiles. LCA represents a powerful method of cluster analysis that can be used to identify patterns of similar responses for categorical indicator variables (e.g., symptom present or symptom absent). The goal is to create a set of exclusive latent classes; that is, to split respondents into groups with homogeneous symptom profiles. This approach has been previously used in bereavement literature to examine symptom profiles of the three most commonly prevalent syndromes in bereaved individuals: MDD, PGD, and PTSD. For instance, Djelantik and colleagues (2017) [18] used this approach to investigate symptom clusters of MDD, PGD, and PTSD in a bereaved community sample, revealing three classes of symptoms: a PGD class (48%), a PGD and PTSD symptom profile class (27%), and a resilient class (25%). Another study applying LCA to a trauma-exposed bereaved sample revealed four classes: a resilient class (13%), a class with prominent PGD and MDD symptoms (23%), a class with PGD and PTSD symptoms (20%), and a class with MDD, PGD, and PTSD symptoms (45%) [19]. A study including a disaster-bereaved sample again revealed three classes as the best fit: a resilient class with low probability of MDD, PGD, and PTSD symptoms (20%), a class characterized only by PGD symptoms (41.8%), and a combined class with a moderate to high probability for the presence of all three symptom clusters (38.2%) [20]. One recent study focusing on a recently bereaved sample examined symptoms of MDD, PGD, and PTSD within the first 6 months of bereavement and revealed again a three-class symptom profile: a low-symptom class (35.4%), a predominantly PGD class (29.8%), and a high-symptom profile class (34.8%) including symptoms of all symptom clusters [21]. For a summary of the main findings of previous latent class research following bereavement, see Figure 1.

While existing research highlights the predominant role of PGD symptoms in bereaved populations, the examination of these symptom clusters in suicide-bereaved individuals remains unclear. The aim of the present study was to identify classes regarding symptoms of MDD, PGD, and PTSD in a German sample of suicide-bereaved individuals by examining patterns of symptom co-occurrence. We additionally aimed to examine possible differences in symptom severity based on loss-related characteristics such as kinship and the period of time since the loss. The examination of symptom clusters may offer important insights for the refinement of psychological treatments for the suicide bereaved.

## 2. Materials and Methods

### 2.1. Participants and Procedure

Participant data were derived from a help-seeking sample, which completed the assessment for participation in a randomized controlled trial assessing the effectiveness of an online group intervention for suicide-bereaved adults [22]. The study protocol was approved by the review board of the Medical School Hamburg. Participant recruitment took place primarily through support organizations for bereaved individuals in Germany, as well as through online advertisements. After signing the informed consent form, participants aged between 18 and 75 years were invited to complete the online screening assessment, followed by a telephone interview conducted by the study coordinators. Participants with acute suicidality, self-harm, current psychosis, alcohol or substance abuse, severe depression (BDI > 35), bipolar disorder, or borderline personality disorder were not eligible for participation in the trial. This resulted in a total number of N = 159 participants who completed the screening assessment.

### 2.2. Variables and Measures

Sociodemographic variables included in our analysis were gender, age of the bereaved, education, and marital status. Loss-related variables comprised the age of the bereaved since the day of loss, and relationship to the deceased.

Due to the limitation of the number of item inclusions in the LCA analysis, a subset of core symptoms for each disorder in correspondence with the diagnostic criteria of the ICD-11 and/or DSM-5 was included. In case of overlap of items between two disorders, items were included in the category which we considered more representative. The items of each questionnaire were dichotomized in 0 (=symptom absent for lower item ratings) and 1 (=symptom present for higher item ratings), based on prior research examining latent class symptom profiles of bereaved individuals [19,23].

Symptoms of depression were assessed with the revised German version of the Beck Depression Inventory (BDI-II; Beck, Steer, & Brown., 1996 [24]; Hautzinger, Keller, Kühner, & Beck, 2009 [25]), assessing depressive symptom severity. Each symptom is formulated as four statements with increasing levels of severity (e.g., sadness/depressed mood: 0 = “I do feel sad”–3 = “I am so sad and unhappy I cannot stand it”). We selected 7 out of 21 BDI-II items in accordance with depressive disorder symptoms in the ICD-11. We included both “worthlessness” and “guilt”, as these symptoms were especially relevant for the suicide-bereaved sample. Furthermore, we excluded the item “concentration difficulty”, as there was an overlap with the scale for posttraumatic stress symptoms, as well as “suicide ideation”, as acute suicidality represented one of the exclusion criteria for participation in the study (for an overview of the included items and the corresponding symptom description, see Table 1). Based on a previously set cut-off from previous research conducting LCA analyses with the BDI-II, each item was considered absent (=0) if rated with 0 or 1, while it was considered present (=1) if rated with 2 or 3 [18].

Symptoms of prolonged grief were assessed with the German version of the Inventory of Complicated Grief (ICG; Prigerson et al., 1995 [26]; ICG-D; Lumbeck, Brandstaetter, & Geissner, 2012 [27]). We selected 8 out of 19 items of the ICG corresponding closely to the diagnostic criteria proposed by Prigerson et al. (2009) [28], allowing a reliable distinction from other mental health disorders (Bellini et al., 2018; Boelen, 2013; Stroebe, Schut, & Van den Bout, 2013 [10,16,29,30]). According to previous studies conducting LCA research using the ICG, we created dichotomized indicator variables for each included item (Djelantik et al., 2017; Lenferink et al., 2017; Zhou et al., 2018 [18,20,31]). Each item was defined as symptom absent (=0) for ratings between 1 = “never” to 2 = “rarely” and was considered as symptom present (=1) for ratings between 3 = “sometimes” to 5 = “always”.

Symptoms of posttraumatic stress were measured with the use of the adapted version of the Impact of Event Scale-Revised (IES-R; Horowitz et al., 1979 [32]; Maercker & Schützwohl, 1998 [33]), which assesses symptoms of PTSD. Items were selected as corresponding to the DSM-5 categories for intrusion symptoms (thoughts, images, dreams), avoidance of thoughts and behavior (reminders, thoughts, numbness), and changes in arousal and reactivity (irritability/anger, concentration difficulties). Aiming for consistency of dichotomization, we set a similar cut-off as described for the measures above. A symptom rating between 0 = “not at all” and 1 = “minimal” was defined as symptom absent (=0) while ratings between 2 = “moderately” and 4 = “extremely” were considered as symptom present (=1).

### 2.3. Data Analysis

The first part of the statistical analysis was conducted in RStudio, version 4.0.0 (RStudio Team, Boston, MA, USA 2020 [34]). No examination of missing data was necessary, as a full adherence at the screening assessment was a prerequisite for participation in the trial. To identify classes of suicide-bereaved individuals with comparable MDD, PGD, and PTSD symptom profiles, the poLCA package was used [35]. The estimation process began with two latent class profiles, while increasing the number of profiles in order to find the optimal number of classes. For the identification of model fit, the following indices were examined: Akaike’s Information Criterion (AIC), Bayesian Information Criterion (BIC), and entropy (classification quality). As indicated in previous research, lower AIC and BIC values and higher entropy values indicate better fit [36]. Furthermore, an additional LCA was conducted, adding important covariates in our model.

## 3. Results

### 3.1. Sociodemograhic Characteristics

The sample consisted of 159 adults (*n* = 142, 89.3% female) with a mean age of 40.62 (SD = 12.67) years. The majority of the sample was in a relationship (*n* = 31, 19.5%) or married (*n*= 45, 28.3%), and had received a degree from vocational school, university or higher (69.8%). The sample included first-degree bereaved relatives (*n* = 102; 64.2%), bereaved spouses or partners (*n* = 36, 22.6%), and bereaved friends, colleagues, or others (*n* = 21, 13.2%). The mean age of the bereaved individual at the time of loss was 41.45 (SD = 17.02) years. The mean time since the loss was 28.18 (SD = 55.77) months. For an overview of all sociodemographic and loss-related characteristics, see Table 2.

### 3.2. Latent Class Analysis

**Figure 2:**
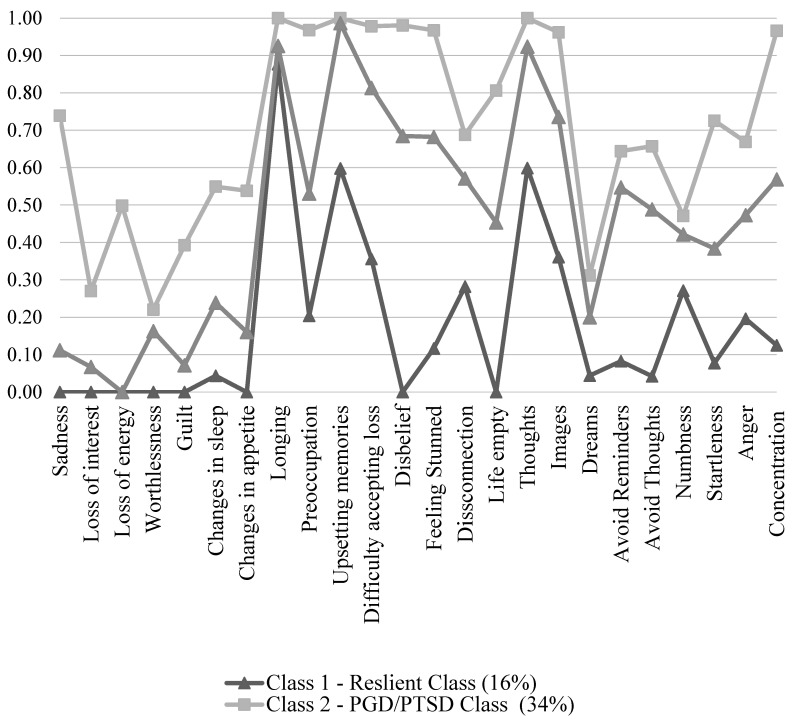
Estimated symptom endorsement profiles for the 3-class model of the latent class analysis (*N* = 159).

The fit indices for the latent class profiles are listed in Table 3. Based on the goodness-of-fit indices, three class solutions appeared adequate with a better fit and interpretability, and were therefore retained. We considered a value of >0.60 as a high probability of symptom endorsement [19,37]. The three-class model revealed one resilient class (class 1; 16% of the sample), one class with a higher probability for some symptoms of PGD and intrusion symptoms of PTSD (class 2; 50% sample), and one class with prominent symptoms of PGD and PTSD (class 3; 34% of the sample). The distinct symptom occurrences are presented in Figure 2.

In class one (resilient class), only the PGD items “longing for the deceased” and “upsetting memories”, as well as the PTSD item “intrusive thoughts”, had a high probability of endorsement. In class two (PGD class), all PGD items except “disconnection” and “life is empty” showed a high endorsement probability, while this was again observed for the intrusion items of PTSD “intrusive thoughts” and “intrusive images”. In class three (combined PGD and PTSD class), all PGD items and PTSD items except “intrusive dreams” and “numbness” had a high endorsement probability. This is the only class in which a symptom of depression showed higher endorsement, though this only applied to the item “sadness”. In general, symptoms of PGD and especially “longing” and “upsetting memories”, similar to the PTSD item “intrusive thoughts”, had a high endorsement probability in all classes, while the probability for endorsement of all depressive symptoms, besides “sadness”, was low.

We extended our analysis by adding important covariates to our LCA analysis. Table 4 depicts differences between the classes regarding sociodemographic (age, gender) and loss-related (kinship, time passed since day of loss) variables between the classes. The first (resilient) class was used as the class of reference. No statistically significant differences between the classes for age, gender, or kinship were found. Time passed since the day of loss was the only variable significantly associated with the class membership. However, this significance only applied to differences between the PGD and the resilient class. Time since loss appeared lower for individuals in the PGD class compared to the resilient class.

## 4. Discussion

To our knowledge, this is the first study employing LCA to explore classes of MDD, PGD, and PTSD symptoms in a suicide-bereaved sample. Half of the examined population showed mainly elevated PGD symptom endorsement, while one-third of the included sample showed high PGD and PTSD symptom endorsement. PGD symptoms had a high endorsement probability for all classes, while no pattern of increased MDD symptom endorsement was revealed. A tendency towards higher endorsement probability for one MDD item appeared, but only in the class characterized by increased combined PGD and PTSD endorsement.

Our findings are in line with previous research focusing on community or trauma-exposed samples showing classes of patients with increased PGD and with combined PGD and PTSD symptom endorsement [18,19]. Based on the findings of this symptom approach, it becomes clear that the targeted treatment of PGD reactions might be especially valuable for the highest proportion of the population. “Longing/ yearning” appears to be the most predominant symptom across all classes, independent of the time passed since the loss [18,23]. Additionally, our findings are in line with research stressing the need to pay attention to treatment options targeting comorbid PGD and PTSD symptoms [18,19,38]. Again, intrusive posttraumatic stress symptoms and in particular “intrusive thoughts” showed high endorsement in all classes. Our findings offer an indication that time since the loss might play a role in PGD symptom reduction, while there is no similar indication for comorbid PGD/PTSD symptoms.

## 5. Conclusions

In conclusion, the assessment for comorbid treatment of PTSD appears crucial for those bereaved through suicide. Similarly, the finding that a subgroup of participants appeared to be affected by combined symptoms of PGD, PTSD, and only to a lesser extent by MDD symptoms, agrees with previous research focusing on bereaved individuals [18,23,39]. Thus, one partial explanation for our findings could be the exclusion of more severe depression. Including severely depressed bereaved individuals might have led to differential findings.

Several limitations should be noted. First, our data are based on self-report questionnaires, leading to strengthened associations between variables. Second, our sample consisted mainly of female, highly educated adults. Future studies should aim to examine larger and more heterogeneous suicide-bereaved samples. Third, there was a wide range regarding the time since bereavement, ranging from less than one month to more than 20 years. A high percentage of participants were bereaved for more than 6 months, which is the prerequisite for a PGD diagnosis [28]. Furthermore, there was not a full correspondence with the diagnostic criteria, in contrast to the ICD-11 and DSM-5 criteria. In our approach, which aimed at minimizing the number of items for the analysis and including only the most characteristic symptoms of each disorder, we intentionally excluded doubled items (e.g., “anger”, “concentration difficulties”) which simultaneously resemble symptoms of more than one clinical diagnosis category. Through including all items, it appears likely that symptom endorsement would increase as the interrelatedness of symptoms would increase [16,17]. Thus, assessing formal diagnoses was beyond the scope of the present study. A replication of our findings on a larger population, while also examining the full spectrum of ICD-11 and DSM-5 criteria, is needed.

Notwithstanding the above-mentioned limitations, this study offers an important first insight into subgroups of those bereaved through suicide, based on a symptom-level approach. This was examined in a sample with a wide range of time elapsed since the loss, and with different levels of kinship or relationship to the deceased. We found three distinct classes of suicide bereavement, with participants appearing to be especially affected by PGD symptoms, commonly combined with the presence of PTSD symptoms, and especially with symptoms of intrusion. In line with previous findings on bereavement, our findings highlight the necessity for concurrent treatment for PGD and PTSD for the suicide bereaved [18]. Taking the traumatic circumstances following a violent death and the exposure when discovering the deceased into account, it is not surprising that this population shows an increased risk for developing PTSD [40]. Moreover, previous research underlines the relatedness of PGD and PTSD, suggesting that the likelihood of yearning for the deceased is higher for individuals who experience death-related intrusive thoughts and memories related to the circumstances of death [41]. This attempt to avoid painful experiences appears to mediate the relationship between the traumatic distress and yearning. Trauma-focused, exposure-based treatments targeting PGD and PTSD symptoms simultaneously, while promoting the development of a comprehensive narrative, could represent effective treatment options for those bereaved through suicide [42,43]. It appears likely that a targeted treatment for PGD and PTSD could lead to simultaneous MDD symptom relief.

## Figures and Tables

**Table 1:** Description of included symptoms of each questionnaire.

	BDI-II Items for MDD Symptoms	ICG-D Items for PGD Symptoms	IES-R Items for PTSD Symptoms	
1	Sadness	Longing/Yearning	Intrusion	Thoughts
2	Loss of interest	Preoccupation		Images
3	Loss of energy	Upsetting memories		Dreams
4	Worthlessness	Difficulty accepting the loss	Avoidance	Avoid reminders
5	Guilt	Disbelief		Avoid thoughts
6	Changes in sleep	Feeling stunned/numb since loss		Numbness
7	Changes in appetite	Disconnection from others	Hypervigilance	Startled
8		Feeling life is empty		Anger
9				Concentration difficulty

**Table 2:** Sociodemographic and loss-related characteristics of the sample.

	*n* (%)	M (SD)	Range
Gender (female)	142 (89.3%)		
Age		40.62 (12.67)	19.0–69.0
Marital status			
in a relationship	31(19.5%)		
married	45 (28.3%)		
single	51 (32.1%)		
divorced	10 (6.3%)		
widowed	22 (13.8%)		
Education			
low	7 (4.4%)		
middle	41 (25.8%)		
high	111 (69.8%)		
Age of bereaved at time of loss		41.45 (17.02)	3.0–68.0
Kinship to deceased			
child	40 (25.2%)		
parent	27 (17.0%)		
sibling	35 (22.0%)		
spouse/partner	36 (22.6%)		
friend/colleague/other	21 (13.2%)		
Time passed since loss (months)		28.18 (55.77)	0.0–312.0
<6 months	62 (39.0%)		
6–12 months	39 (24.5%)		
13–24 months	28 (17.6%)		
>24 months	30 (18.9%)		

**Table 3:** Goodness-of-fit criteria.

	2 Classes	3 Classes	4 Classes	5 Classes
Number of estimated parameters	49	74	99	124
Residual degrees of freedom	110	85	60	35
AIC	3870.78	3782.93	3774.32	3761.15
BIC	4021.15	4010.03	4078.14	4141.70
Likelihood ratio	2171.95	2034.11	1975.49	1912.33
Entropy	0.87	0.90	0.93	0.89

Note. AIC = Akaike information criterion; BIC = Bayesian information criterion.

**Table 4:** Latent class model with analysis of covariance.

	B	SE	*t*-Value	*p*-Value
**PGD/PTSD vs. resilient class**				
Age	−0.01	0.04	−0.39	0.70
Gender	1.32	0.97	1.36	0.18
Kinship	0.05	0.25	0.19	0.85
Time since the loss	−0.40	0.50	−0.80	0.42
**PGD vs. resilient class**				
Age	−0.01	0.04	−0.25	0.81
Gender	1.53	1.42	1.07	0.29
Kinship	0.49	0.31	1.60	0.12
Time since the loss	−1.34	0.54	−2.50	0.01

Note. B = Beta; SE = Standard Error; PGD = Prolonged Grief Disorder; PTSD = Posttraumatic Stress Disorder.

## Data Availability

Data available on request due to privacy/ethical restrictions.
